# Glucocorticoid Receptor Antagonist Administration Prevents Adrenal Gland Atrophy in an ACTH-Independent Cushing's Syndrome Rat Model

**DOI:** 10.1155/2019/8708401

**Published:** 2019-02-19

**Authors:** Atsushi Yasuda, Toshiro Seki, Yoshie Kametani, Masahiro Koizumi, Natsumi Kitajima, Masayuki Oki, Masami Seki, Takatoshi Kakuta, Masafumi Fukagawa

**Affiliations:** ^1^Division of Nephrology, Endocrinology and Metabolism, Department of Internal Medicine, Tokai University School of Medicine, Kanagawa 259-1193, Japan; ^2^Department of Molecular Life Science, Division of Basic Medical Science, Tokai University School of Medicine, Kanagawa 259-1193, Japan; ^3^Division of General Internal Medicine, Department of Internal Medicine, Tokai University School of Medicine, Kanagawa 259-1193, Japan; ^4^Seirei Numazu Hospital, Shizuoka 410-8555, Japan

## Abstract

ACTH-independent Cushing's syndrome (CS) is mainly caused by cortisol-secreting adrenocortical tumours. It is well known that secondary adrenal insufficiency occurs after surgical resection of these tumours. In this regard, impaired adrenocortical function is likely induced by atrophy of the residual adrenal tissue as a result of chronic suppression by the low ACTH levels of the hypercortisolism state. Therefore, we considered the prevention of adrenal atrophy as a method for preventing postoperative adrenal insufficiency. On the basis of these findings, we hypothesized that the use of a glucocorticoid receptor (GR) antagonist before surgery in ACTH-independent CS would rapidly activate the hypothalamic-pituitary-adrenal (HPA) axis and residual adrenal function. We thus examined adrenal function in a dexamethasone- (DEX-) induced CS rat model with or without mifepristone (MIF). In this study, MIF-treated rats had elevated plasma ACTH levels and increased adrenal weights. In addition, we confirmed that there were fewer atrophic changes, as measured by the pathological findings and mRNA expression levels of corticosterone synthase *CYP11B1* in the adrenal glands, in MIF-treated rats. These results indicate that MIF treatment prevents the suppression of the HPA axis and the atrophy of the residual adrenal tissue. Therefore, our study suggests that preoperative GR antagonist administration may improve residual adrenal function and prevent postoperative adrenal insufficiency in ACTH-independent CS.

## 1. Introduction

ACTH-independent Cushing's syndrome (CS) is mainly caused by cortisol-secreting adrenocortical tumours [1]. The hypercortisolism of CS causes cardiovascular disease, glucose and lipid metabolism disorders, infectious disease, bone metabolism disorders, and a markedly reduced quality-of-life prognosis [1]. The first-line treatment for this disease is adrenal surgery; nevertheless, secondary adrenal insufficiency develops after surgical resection of cortisol-secreting tumours in most cases [2]. It is likely that more than several months or years, in some cases, are required for the functional recovery of the remaining adrenal tissue [3]. For postoperative patients with ACTH-independent CS, adrenal insufficiency reduces their quality of life, and there exists a possibility that the adrenocortical function of these patients will remain permanently impaired. Accordingly, these patients require lifelong steroid replacement therapy.

As recent improvements and the widespread use of imaging studies have made the detection of bilateral adrenal tumours easier, we foresee that diagnoses of ACTH-independent CS due to bilateral cortisol-producing tumours will increase even further in the near future. Our group reported that adrenal venous sampling is useful for obtaining a definitive diagnosis for ACTH-independent CS with bilateral adrenal tumours [4]. Additionally, we reported a case of bilateral cortisol-producing adenomas that caused CS in a patient who underwent laparoscopic adrenalectomy, namely, a total left and partial right adrenalectomy [5]. In this case, the functional recovery of the remaining adrenal tissue was not detected. From these results, it was noted that adrenal functional recovery was difficult, especially after an adrenal-preserving surgery for the bilateral adrenocortical adenoma cases compared with the unilateral cases due to atrophied adrenal glands.

The main pathophysiological mechanism of adrenal insufficiency is reported to be that the residual adrenocortical tissue becomes atrophied because of chronic suppression of the hypothalamic-pituitary-adrenal (HPA) axis in the hypercortisolism state [6]. Chronic hypercortisolism suppresses the activity of hypothalamic CRH-producing cells, and glucocorticoids have an inhibitory effect on these cells, which then causes a decrease in ACTH secretion [7]. The reduction in ACTH stimulation leads to the atrophy of the residual adrenal cortex, and improvements in the HPA axis generally require several months to years to achieve [6].

Several medication protocols exist for CS, and these include a GR antagonist and adrenal steroidogenesis inhibitors (ketoconazole, metyrapone, mitotane, and etomidate) [8, 9]. Mifepristone (MIF), 11*β*-(P-(dimethylamino)phenyl)-17*β*-hydroxy-17-(1-propynyl)estra-4,9-dien-3-one)), is a progesterone receptor antagonist that is used for early termination of pregnancy and has GR antagonist activity at high concentrations, and its binding affinity for the GR is stronger than that of cortisol or dexamethasone (DEX) [10, 11]. Additionally, a previous study suggested that the receptor-blocking effect of MIF to treat hypercortisolism produced significant improvements in various disease aspects, including insulin resistance, blood pressure control, psychiatric symptoms, and quality of life when administered to patients with CS [12].

Therefore, we proposed the following hypothesis for the effect of MIF. In the preoperative management of ACTH-independent CS, as expected with GR antagonism, the HPA axis is rapidly activated postoperatively due to the use of MIF, with the ACTH levels rising appropriately, and ACTH stimulates the remaining adrenal tissue to grow. Accordingly, the function of the remaining adrenal tissue may be easily preserved after surgery. In addition, the preoperative administration of MIF may increase the control of diabetes mellitus and hypertension and improve the quality of life in patients with CS. We thus evaluated adrenal function in a DEX-induced CS rat model with or without MIF treatment and verified that the residual adrenal function was preserved by the administration of MIF.

## 2. Materials and Methods

### 2.1. Animals

Male Sprague-Dawley rats, aged 10 weeks (400-450 g), were purchased from Charles River Laboratories Japan (Atsugi, Japan). The rats were housed in individual cages under environmentally controlled conditions (light from 8 AM to 8 PM, temperature 23 ± 2°C, and rat chow *ad libitum*). All experimental protocols were performed in accordance with the National Institutes of Health (NIH) Guide for the Care and Use of Laboratory Animals and approved by the Institutional Animal Care and Use Committee of Tokai University (permit number, 184005). All efforts were made to minimize the number of animals used and the suffering caused by the procedures. One week after arrival, the rats were randomly divided into each group (*n* = 5). The experiment began on day 0, and drug administration was performed beginning on day 1.

### 2.2. Experimental Protocol

#### 2.2.1. Experiment 1

In the first experiment, we investigated whether the administration of MIF in an ACTH-independent CS rat model could prevent the suppression of the HPA axis. DEX was obtained from Wako Pure Chemical Industries (Osaka, Japan) and dissolved in propylene glycol (Wako Pure Chemical Industries). DEX was then administered subcutaneously at a dose of 5 *μ*g/100 g body weight to the rats in all four groups for 14 days. MIF was supplied by Tokyo Chemical Industry (Tokyo, Japan). On days 8-14, the second (DEX + MIF3), third (DEX + MIF6), and fourth (DEX + MIF9) groups ingested 3 mg/100 g, 6 mg/100 g, and 9 mg/100 g body weight MIF, respectively, which was suspended in water containing 1% carboxymethyl cellulose (Wako Pure Chemical Industries) and 0.20% polysorbate 80 (Sigma-Aldrich Japan, Tokyo, Japan). The first (DEX) group ingested water containing 1% carboxymethyl cellulose with 0.20% polysorbate 80 as a control. The blood samples were collected from the tail veins after fasting for 12 hours to measure the ACTH levels on days 0, 7, and 14 after the initiation of the experiment.

#### 2.2.2. Experiment 2

**Figure 1:**
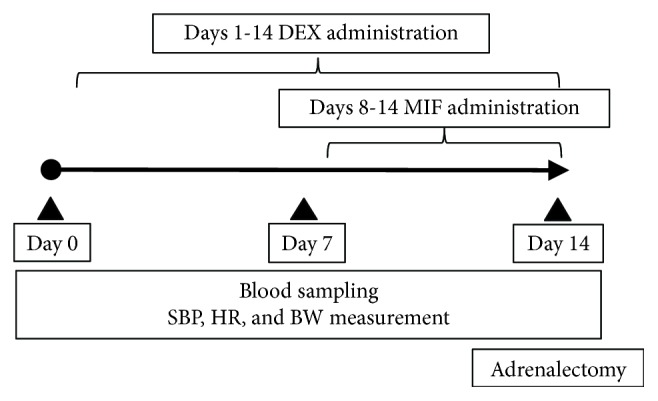
Experimental protocol. The protocol of Experiment 2 is shown in the figure.

We determined the appropriate MIF dose based on the results of the first experiment. In the second experiment, we tested whether the administration of MIF in the rat model could preserve the function of residual adrenal tissue as shown in the protocol (Figure 1). DEX was administered subcutaneously at a dose of 5 *μ*g/100 g body weight to the second (DEX) and third (DEX + MIF) groups for 14 days. The first (control) group received propylene glycol subcutaneously as a control. On days 8-14, the third group ingested 6 mg/100 g body weight MIF that was suspended in water containing 1% carboxymethyl cellulose with 0.20% polysorbate 80. The first and second groups ingested water containing 1% carboxymethyl cellulose with 0.20% polysorbate 80 as a control. Systolic blood pressure (SBP), heart rate (HR), and body weight (BW) were measured on days 0, 7, and 14 after starting the experiment. In addition, blood samples were collected from the tail veins after fasting for 12 hours to measure the levels of ACTH, electrolytes, and blood glucose on days 0, 7, and 14 after starting the experiment.

### 2.3. SBP Measurement

SBP and HR were measured with the tail cuff method using the Noninvasive Blood Pressure Monitor for Mice and Rats (MK-2000; Muromachi Kikai, Tokyo, Japan). Rats were placed on a heated platform (37°C) under anaesthesia with 1.5-2% isoflurane. The cuff contained a photoelectric pulse detector and was placed on the tail and inflated by the pump. The pressure inside the cuff and the pulse curve were registered, and the pressure at which the first oscillation appeared during the reduction of the cuff pressure was the SBP of the animal. Three blood pressure measurements were obtained for each rat and averaged.

### 2.4. Surgery

Fourteen days after the initiation of the treatment, all rats were anaesthetized with isoflurane, their abdomens were dissected, and their adrenal glands on both sides were removed. The adrenal glands were cleaned and weighed. The left gland was used for histochemical analysis, and the right adrenal gland was used for the real-time PCR analysis as described below.

### 2.5. ACTH ELISA

Blood samples were collected in plastic tubes (Scientific Specialities Inc., Lodi, CA, USA) containing EDTA and protease inhibitor cocktail (Sigma-Aldrich, Japan) and placed on wet ice. The sample was immediately sent to the laboratory and then centrifuged for 6 min (4°C), and 500 *μ*L of plasma was transferred to another microfuge tube. Rat ACTH levels were determined using a commercially available enzyme-linked immunosorbent assay (ELISA) kit (MD Biosciences, St. Paul, Minnesota) with reference to the previous report [13].

### 2.6. Histological Analysis

Each sample was fixed in formalin and embedded in paraffin, and the sliced sections were stained with haematoxylin and eosin (HE) according to a standard method. Atrophy of the adrenal cortex was quantitatively evaluated by comparing the area of the adrenal cortex to medulla ratios (corticomedullary area ratios) using five tissue sections from the central part of the adrenal glands. The measurements of the areas of the adrenal cortexes and medullas were performed according to a previous report [14]. The HE-stained sections were photographed using a microscope (BX63; Olympus, Tokyo, Japan) that was equipped with a digital camera (DP73; Olympus). The areas of the cortex and medulla were determined using ImageJ analysis software (NIH, Bethesda, Maryland, USA).

### 2.7. Determination of CYP11B1 and CYP11B2 mRNA Levels

Gene expression of the adrenal tissues was analysed by using real-time PCR. The adrenal tissues were used for a subsequent analysis of the expression of mRNA for corticosterone synthase 11*β*-hydroxylase (CYP11B1) and aldosterone synthase (CYP11B2). The adrenal glands were removed from the rats, cleared of their adherent fat, and immediately stored in RNAlater solution (Thermo Fisher Scientific, Kanagawa, Japan) for 24 hours at 4°C. The tissues were frozen and stored at -80°C for RNA isolation. Total RNA was extracted from the whole adrenal gland according to a standard method (PrimeScript RT reagent, Takara Bio, Shiga, Japan), and 2 *μ*g was used per assay. The amount of specific RNA was determined by performing real-time RT-PCR according to the recommendations of the manufacturer (Applied Biosystems, Foster City, California, USA [15]). Specific primers for the PCR reactions were designed according to previously published *CYP11B1* and *CYP11B2* primer sequences [16]. The *CYP11B1* primers had the following sequences: sense primer, GTCTATAAACATTCAGTCCAA and antisense primer, ATCTCGGATATGACACTCC. The *CYP11B2* primers had the following sequences: sense primer, GGATGTCCAGCAAAGTCTC and antisense primer, ATTAGTGCTGCCACAATGC. Quantification of the expression of the target gene in relation to *β* actin (as housekeeping gene) was performed employing the comparative (ΔΔ) Ct method.

### 2.8. Statistical Analysis

All data are expressed as the mean ± SEM of multiple experiments. Statistical analyses were performed using a one-way analysis of variance (ANOVA) followed by a Tukey's post hoc test. Statistical significance was confirmed with a *P* value < 0.05.

## 3. Results

### 3.1. Experiment 1

The first (DEX) group showed a tendency towards decreased plasma ACTH levels following DEX administration on day 7 and day 14 (10.97 ± 5.73 and 7.20 ± 4.15 pg/mL, respectively). The plasma ACTH levels of the second (DEX + MIF3), third (DEX + MIF6), and fourth (DEX + MIF9) groups showed a tendency to increase after MIF administration compared to the levels of the first (DEX) group on day 14 (9.44 ± 6.11, 20.30 ± 5.16, 31.81 ± 9.27, and 7.20 ± 4.15 pg/mL, respectively). There tended to be a plasma ACTH dose-related response to MIF, suggesting that higher levels of MIF would release the suppression of the HPA axis. In the DEX + MIF6 group and the DEX + MIF9 group, these plasma ACTH levels showed a significant increase compared to the DEX group on day 14 (*P* < 0.05). However, since an excessive reduction in blood pressure was observed in the DEX + MIF9 group during the administration period (data not shown), we determined that the DEX + MIF6 group was administered the appropriate dose of MIF. Based on the above results, we chose 6 mg/100 g MIF for Experiment 2.

### 3.2. Experiment 2

**Figure 2:**
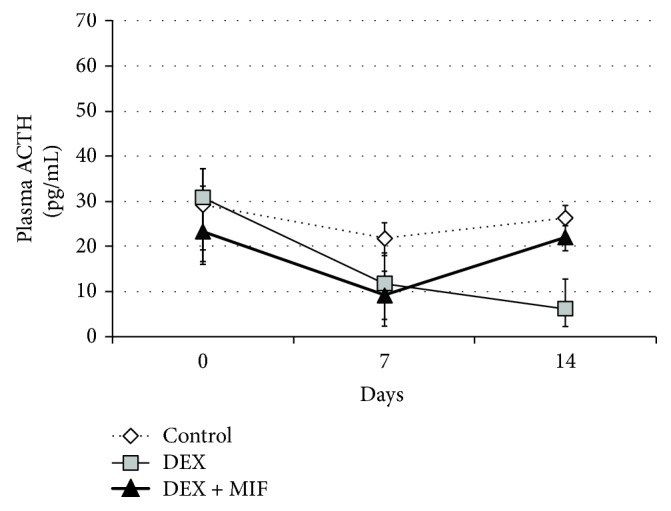
Plasma ACTH levels in Experiment 2. The plasma ACTH levels of rats from each group were measured with the condition that was determined in Experiment 1. The level is shown in pg/mL.

The plasma ACTH levels after MIF administration in the DEX + MIF group were significantly higher than those in the DEX group on day 14 (22.08 ± 3.60 and 6.09 ± 5.30 pg/mL, respectively, *P* < 0.05; Figure 2).

**Figure 3:**
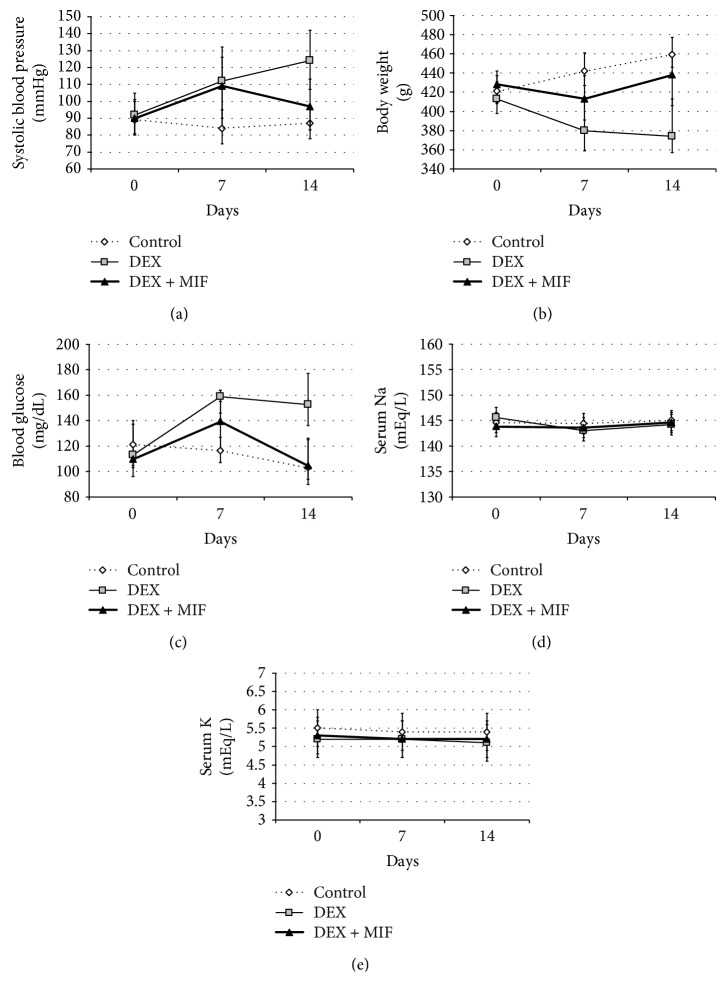
Effect of DEX and MIF treatment on systolic blood pressure, body weight, blood glucose, and serum sodium (Na) and potassium (K). (a) Systolic blood pressure, the systolic blood pressure values of rats in each group are shown in mmHg. (b) Body weight, the body weights of rats from each group are shown in g. (c) Blood glucose level, the blood glucose levels in the sera of rats of each group are shown in mg/dL. ^∗^*P* < 0.05, DEX vs. DEX + MIF and control. (d) Serum Na: the sodium levels in the sera of rats from each group are shown in mEq/L. (e) Serum K: the potassium levels in the sera of rats from each group are shown in mEq/L.

SBP tended to increase in the DEX group, whereas in the DEX + MIF group, a suppression of the SBP elevation was observed in the period after MIF administration (124.3 ± 17.2 and 97.3 ± 15.3 mmHg, respectively; Figure 3(a)). Regarding BW, the decrease during the MIF administration period tended to be suppressed in the DEX + MIF group compared to that of the DEX group on day 14 (438.0 ± 26.1 and 374.3 ± 27.7 g, respectively; Figure 3(b)). In the DEX group, blood glucose was significantly increased, while in the DEX + MIF group blood glucose level was increased until day 7, but it decreased to the same level as that of the control group after 14 days (152.5 ± 20.5, 104.5 ± 16.1, and 103.0 ± 17.5 mg/dL, respectively, *P* < 0.05; Figure 3(c)). Regarding serums sodium (Na) and potassium (K), the three groups showed approximately similar ranges (Figures 3(d) and 3(e)).

**Figure 4:**
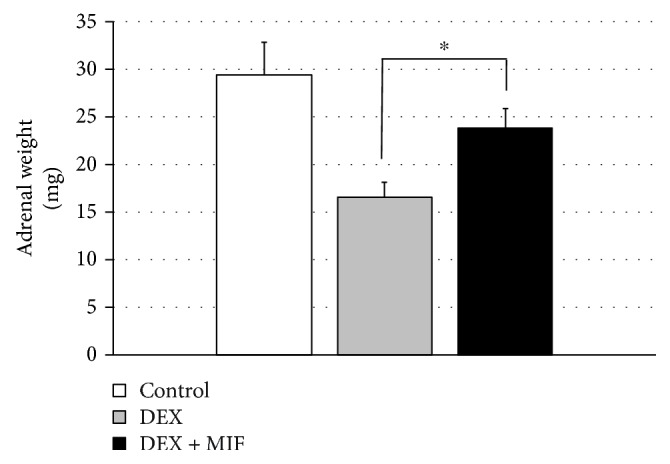
Effects of DEX and MIF treatment on adrenal weights. The diagram panel shows the weights of each rat that was treated with DEX, DEX + MIF, and control. ^∗^*P* < 0.05, DEX vs. DEX + MIF.

In the DEX group, the adrenal weight was decreased, whereas the adrenal weight was significantly increased in the DEX + MIF group after MIF administration compared to that of DEX alone, although the recovery was not complete (16.5 ± 1.6 and 23.8 ± 2.0 mg, respectively, *P* < 0.05; Figure 4).

**Figure 5:**
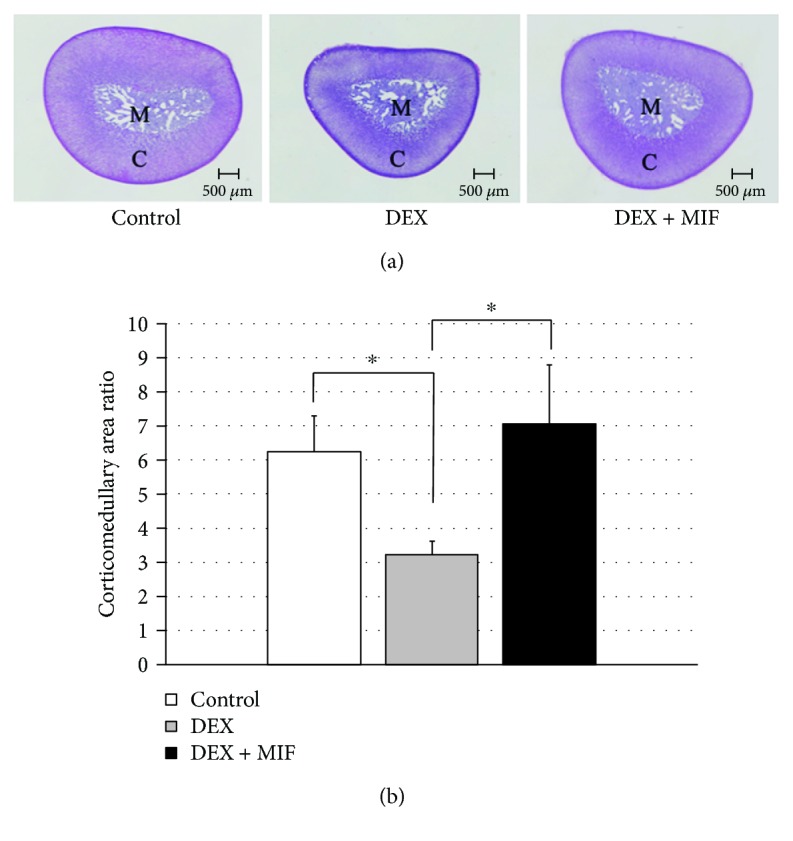
Corticomedullary area ratio of adrenal gland tissues. (a) Representative images of the control (left panel)-, DEX (middle panel)-, and DEX + MIF (right panel)-treated adrenal gland tissues stained with haematoxylin and eosin are shown. C, adrenal cortex; M, adrenal medulla. The bars represent 500 *μ*m. (b) The adrenal cortex and medulla areas were measured, and the cortex/medulla ratios for each group are shown. ^∗^*P* < 0.05, control vs. DEX and DEX vs. DEX + MIF.

HE stain of the tissue sections was performed to examine the ratio of the medulla in the centre of the adrenal gland to the cortex on the outside of the gland (Figure 5(a)). The corticomedullary area ratios were significantly increased in the control group and in the DEX + MIF group compared to that in the DEX group (6.24 ± 1.06, 7.06 ± 1.74, and 3.24 ± 0.37, respectively, *P* < 0.05; Figure 5(b)).

**Figure 6:**
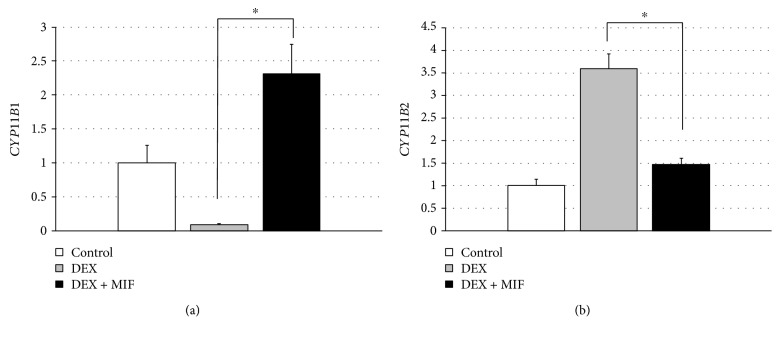
Comparison of *CYP11B1* and *CYP11B2* mRNA expression in the rat adrenal gland after the treatment with DEX and MIF. The ratios were calculated based on the real-time RT-PCR analysis. Quantification of the expression of the target gene was performed in relation to *β* actin as housekeeping gene. The mRNA expression ratios of *CYP11B1* (a) and *CYP11B2* (b) of the adrenal grand from rats in the DEX, DEX + MIF, and control groups were calculated relative to the control group, which had a value that was set to 1. ^∗^*P* < 0.05, DEX vs. DEX + MIF.

The RT-PCR results indicated that the expression level of *CYP11B1* was significantly decreased by DEX administration, and *CYP11B1* expression was significantly increased by DEX and MIF administration (0.09 ± 0.02 and 2.31 ± 0.44, respectively, *P* < 0.05; Figure 6(a)). The expression level of *CYP11B2* was decreased in the DEX + MIF group compared to that of the DEX group (1.47 ± 0.13 and 3.59 ± 0.33, respectively, *P* < 0.05; Figure 6(b)).

## 4. Discussion

In this study, we hypothesized that the use of a GR antagonist (MIF) before surgery in ACTH-independent CS would rapidly activate the HPA axis and residual adrenal function. Based on this hypothesis, we evaluated adrenal function in a CS rat model with or without MIF treatment and verified that the residual adrenal function was preserved by the administration of MIF. First, we confirm that our rat system is appropriate as an ACTH-independent CS model. In the DEX group, suppression of ACTH levels, elevated SBP, and elevated blood glucose were observed. These symptoms are comparable with the state of hyperglucocorticoidaemia in ACTH-independent CS [1]. On the other hand, MIF-treated rats showed elevated plasma ACTH levels, and MIF treatment also prevented the increase in blood pressure and improved hyperglycaemia, as shown in a previous study of patients with CS [12]. These findings suggest that our ACTH-independent CS rat model is appropriate to verify the improvements in residual adrenal function that were induced by the administration of MIF.

The second question of this study is how to correctly estimate adrenal atrophy. The main pathophysiological mechanism of adrenal atrophy has been reported as the chronic suppression of the HPA axis in the hypercortisolism state [6]. The suppression of ACTH secretion has been reported to cause atrophy of zona fasciculate cells, but the activation of steroidogenic enzymes does not occur; thus, steroid secretion is decreased, and apoptotic cells are observed [17, 18]. Based on these reports, we evaluated the weight of the adrenal gland, the corticomedullary area ratio, and the mRNA expression for corticosterone synthase *CYP11B1*. In this study, the adrenal weight and the corticomedullary area ratio were significantly increased in MIF-treated rats. Furthermore, the mRNA expression levels of *CYP11B1* in the adrenal glands were significantly increased after MIF administration. Our study correctly presented that the administration of MIF suppresses adrenal gland atrophy and prevents the functional deterioration of the adrenal gland. To our knowledge, the use of MIF for ACTH-independent CS to prevent adrenal insufficiency after surgery has been reported in clinical case [19]. However, no detailed study has been performed to investigate adrenal atrophy in an ACTH-independent CS rat model with or without MIF treatment. Thus, our report could provide important information for a new therapy for reducing adrenal atrophy and improving adrenal function in ACTH-independent CS.

A third question of our study is whether our animal experiments can be clinically useful. A total of 5-10% of all patients with hypertension have endocrine factors as the cause of secondary hypertension, and these factors include primary aldosteronism, pheochromocytoma, and CS [20]. Most cases of ACTH-independent CS are associated with unilateral adrenocortical adenoma, and until recently, bilateral cortisol-secreting tumours have been considered to be a rare cause of ACTH-independent CS. However, with the future advances in medical technology, diagnoses of CS due to bilateral cortisol-secreting tumours may increase. Currently, there is no consensus regarding the appropriate treatment for patients with these cases. Few cases of bilateral adrenal-preserving surgery have also been reported. In addition, little is known about how much residual adrenal tissue is required for adequate adrenal function, given that the bilateral adrenal-preserving surgery does not necessarily preserve normal adrenocortical function. In this regard, our new strategy of the preoperative administration of MIF could preserve adrenal function after a partial adrenalectomy for CS due to bilateral cortisol-secreting tumours.

The selection of drugs for the preoperative treatment of ACTH-independent CS was considered from the viewpoint of the preservation of adrenal function after surgery as follows. Currently, there are several medications for CS, and adrenal steroidogenesis inhibitors such as mitotane and metyrapone are used to treat this disease [21]. Mitotane is a dichlorodiphenyltrichloroethane (DDT) derivative that acts on the adrenal mitochondria, exhibiting adrenocortical injury, and causing irreversible damage to the adrenal cortex [22]. Furthermore, metyrapone inhibits 11*β* hydroxylase and inhibits hormone synthesis in the adrenal cortex [23]. The effects on the adrenal cortex are reversible, and metyrapone is often used as a medication for CS. In contrast, MIF inhibits the GR and promotes the secretion of ACTH. The ACTH receptor stimulates the adrenal cortex, and hormone synthesis in the adrenal cortex is maintained [24]. These previous findings suggested that the actions of these drugs are different in terms of the hormone synthesis in the adrenal cortex; in this regard, we determined that the use of MIF for reducing adrenal atrophy and improving adrenal function is more reasonable.

Regarding the dose of drug used in this experiment, we referred to past reports of the amount and period that the drug is actually used in humans with CS [12, 19]. The human dose was converted to the rat dose with reference to the weight and life span of the rats, and the dosage of MIF and its administration period were calculated. Moreover, the dose was adjusted so as not to cause adverse effects due to an excessive amount because previous reports showed that side effects appeared at more than 10 mg/100 g body weight in a previous experiment in which MIF was administered to monkeys to evaluate its toxicity [25]. Recently, a GR antagonist other than MIF (CORT 125134) that also specifically acts on the GR has been approved, and it seems that this drug could also be expected to be an effective treatment; basically, we thought that the method and concept of this experiment were appropriate. Furthermore, the previous report suggests that histone deacetylase inhibition (HDACi) suppresses GR transcription, and improves hypertension and hyperglycemia in a model of CS [26]. Therefore, HDACi is considered to have an effect of inhibiting the action of GR as well as MIF and is expected as a very useful drug for CS treatment in the future.

Additionally, we considered the molecular mechanism of the treatment that was used as medicine in this study. The CYP11B1 and CYP11B2 enzymes belong to the cytochrome P450 family of enzymes and are involved in the synthesis of adrenocortical hormones [27]. CYP11B1 is located in the adrenal zona fasciculata and is involved in the synthesis of corticosterone, whereas CYP11B2 is located in the adrenal zona glomerulosa and is involved in aldosterone synthesis [27]. The expression of *CYP11B1* has been shown to be increased by stimulation with ACTH, whereas the expression of *CYP11B2* has been shown to increase with sodium loss and to decrease with prolonged ACTH stimulation [28]. Our results also showed increased expression of *CYP11B1* and decreased expression level of *CYP11B2* with ACTH elevation in the MIF administration group, and our results showed opposite trends in the absence of MIF administration. These results were consistent as a function of MIF administration. However, the GR acts to mediate the actions of glucocorticoids and is widely distributed to various tissues [29]. MIF is a competitive inhibitor of the GR and causes an antiglucocorticoid effect. The administration of glucocorticoids has been shown to cause receptor downregulation [30]. For this reason, the continued administration of MIF can further prompt the release of ACTH suppression in the pituitary. When MIF acts on GRs, both an improvement in the residual adrenal function after ACTH-independent CS surgery and the control of diabetes mellitus and hypertension due to hyperglucocorticoidaemia are expected. In this experiment, an appropriate amount of MIF suppressed the rise in blood pressure due to steroid signalling, further preventing catabolism and weight loss, and the increases in blood glucose levels were also appropriately suppressed by MIF administration. Therefore, we considered MIF administration to be suitable for the preoperative treatment of ACTH-independent CS.

Finally, the validity of the experimental system was evaluated, and we examined the method of measuring ACTH in this experiment. The influence of the stress response on ACTH that could occur as a result of pain stimulation during blood collection has been previously described [31]. In this experiment, from the viewpoint of animal welfare, as in the previous report, blood was collected from the tail vein while removing stress through anaesthesia [32]. In another study, it was shown that the effect of anaesthesia on ACTH was not significant, and we performed ACTH measurements under anaesthesia [33]. Regarding ACTH, the rat and human ACTH amino acid sequence is the same at sites 1-39, but there are two different sites. For the ACTH assay that was used in this study, ACTH measurement was performed with a kit using an antibody that was validated for its measurement in rats with past studies [13, 34]. Additionally, the evaluation of SBP was discussed. There is a report that shows that abnormal SBP values tend to occur under anaesthesia or apnoea [35]. However, it is difficult to measure the blood pressure of a moving rat unless it is under anaesthesia. For this reason, blood pressure measurements were performed with reference to a previous report that was conducted under anaesthesia [36, 37]. In these studies, isoflurane anaesthesia has little effects on blood pressure, so it is recommended for use in cardiovascular evaluations. In addition, since the rats in each group were under the same anaesthesia condition, we thought that the SBP values under anaesthesia would be comparable parameters in this study.

## 5. Conclusions

In summary, we examined adrenal function in a DEX-induced CS rat model with or without MIF treatment and verified that the residual adrenal function was preserved by MIF administration. MIF-treated rats showed elevated plasma ACTH levels, increased adrenal weight, normal blood pressure levels, and improved hyperglycaemia. Moreover, MIF-treated rats showed fewer atrophic changes in the pathological analysis, and the mRNA expression levels of *CYP11B1* in the adrenal glands were even further increased compared with those after DEX administration. These results suggest that MIF treatment may prevent the suppression of the HPA axis. Preoperative MIF administration may improve residual adrenal function in ACTH-independent CS.

## Data Availability

The data used to support the findings of this study are available from the corresponding author upon request.
